# Zika virus infection confers protection against West Nile virus challenge in mice

**DOI:** 10.1038/emi.2017.68

**Published:** 2017-09-20

**Authors:** Ángela Vázquez-Calvo, Ana-Belén Blázquez, Estela Escribano-Romero, Teresa Merino-Ramos, Juan-Carlos Saiz, Miguel A Martín-Acebes, Nereida Jiménez de Oya

**Affiliations:** 1 Department of Biotechnology, Instituto Nacional de Investigación y Tecnología Agraria y Alimentaria (INIA), Madrid 28040, Spain

**Keywords:** antibodies, flavivirus, humoral immune response, protection, West Nile virus, Zika virus

## Abstract

Flaviviruses are RNA viruses that constitute a worrisome threat to global human and animal health. Zika virus (ZIKV), which was initially reported to cause a mild disease, recently spread in the Americas, infecting millions of people. During this recent epidemic, ZIKV infection has been linked to serious neurological diseases and birth defects, specifically Guillain-Barrè syndrome (GBS) and microcephaly. Because information about ZIKV immunity remains scarce, we assessed the humoral response of immunocompetent mice to infection with three viral strains of diverse geographical origin (Africa, Asia and America). No infected animals showed any sign of disease or died after infection. However, specific neutralizing antibodies were elicited in all infected mice. Considering the rapid expansion of ZIKV throughout the American continent and its co-circulation with other medically relevant flaviviruses, such as West Nile virus (WNV), the induction of protective immunity between ZIKV and WNV was analyzed. Remarkably, protection after challenge with WNV was observed in mice previously infected with ZIKV, as survival rates were significantly higher than in control mice. Moreover, previous ZIKV infection enhanced the humoral immune response against WNV. These findings may be relevant in geographical areas where both ZIKV and WNV co-circulate, as well as for the future development of broad-spectrum flavivirus vaccines.

## INTRODUCTION

Flaviviruses constitute a group of arboviruses that often represent a worrisome threat to global human and animal health. For example, since the introduction of West Nile virus (WNV) to the United States in 1999, the virus has spread across the country, where it is now considered endemic, and has caused thousands of human deaths. Similarly, WNV outbreaks are increasing in number, frequency, and severity in Europe, causing a considerable number of neuroinvasive cases in animals and humans, with hundreds of human and horse deaths across the continent.^1, 2^

More recently, the introduction and explosive spread of Zika virus (ZIKV) in the Americas has resulted in the infection of millions of people.^3^ ZIKV infection had initially been characterized as causing a mild disease, with sporadic reports of an association with Guillain-Barrè syndrome (GBS).^3, 4, 5^ However, since the end of 2015, an increase in the number of GBS-associated cases and an astonishing number of microcephaly cases in fetuses and infants in Brazil have been linked to ZIKV infection, raising serious worldwide health and social concerns.^3, 4, 5, 6^

Currently, information regarding the pathogenicity and cross-reactive immunity of ZIKV is limited, in part due to the lack of an accurate small animal model. Non-human primates can be used,^7, 8, 9, 10, 11^ but in many instances, ethical and cost reasons discourage their utilization. Early ZIKV studies were based on the inoculation of mouse-adapted viral strains and were mostly conducted by direct intracranial inoculation of the virus and/or the use of juvenile animals.^12, 13, 14, 15^ Hence, no accurate small animal model for ZIKV infection is currently available. However, both immunodeficient^16, 17, 18, 19, 20, 21, 22^ and immunocompetent mice^11, 23, 24^ are proving to be useful for the study of the pathogenesis and humoral responses elicited by ZIKV.

Antibody-mediated immunity is considered a major player in the protection against flavivirus infections,^25^ including ZIKV infection.^26^ Antibodies elicited against these viruses are often cross-reactive with other related flaviviruses; however, while they sometimes confer cross-protection, in other cases harmful consequences are observed due to an antibody-dependent enhancement (ADE) effect.^27^ This, together with the high reported antibody prevalence in ZIKV-infected populations,^3, 28, 29^ may have special relevance in areas where different flaviviruses co-circulate. Indeed, the relationships between the immune response after ZIKV and subsequent or previous Dengue virus (DENV) infection, endemic in areas of central and South America, are being now assessed.^30, 31, 32, 33^ In this study, with the potential colonization of new territories by ZIKV, we explored the capability of this new invader to induce protection against WNV.

## MATERIALS AND METHODS

### Ethics statement

All animals were handled in strict accordance with the guidelines of the European Community 86/609/CEE. The protocols were approved by the Committee on Ethics of animal experimentation of our Institution (INIA’s permit numbers 2016-006 and 2017-008). All experiments with infectious viruses were conducted in biosafety level 3 facilities.

### Viruses

ZIKV strains of American (PA259459) and Asian (FSS13025) origin were kindly provided by Dr R. B. Tesh (World Reference Center for Emerging Viruses and Arboviruses, WRCEVA) and a strain of African origin (MR766) by Dr A. Vázquez (Instituto de Salud Carlos III, ISCIII). Vesicular stomatitis virus (VSV) Indiana strain was kindly provided by Dr Rafael Blasco (Department of Biotechnology, INIA). ZIKV strains, VSV, and a WNV NY99 strain^34^ were propagated and titrated on Vero-81 cells (ATCC CCL-81, Manassas, VA, USA) as described.^35^ ZIKV strains were partially sequenced (Macrogen Europe; Amsterdam, The Netherlands) using specific primers available upon request.

### Mice

Groups (*n*=10–20) of 8-week-old Swiss albino CD1 male mice were intraperitoneally (i.p.) inoculated with 5 × 10^5^ plaque-forming units (pfu)/mouse of the African, American and Asian ZIKV strains, or VSV, in 100 μL of Eagle’s minimal essential medium (EMEM) (BE12-125F, Lonza, Verviers, Belgium) as the vehicle. As a control, a group of CD1 mice was inoculated with vehicle alone. Mice were i.p. challenged with 10^4^ pfu/mouse of a neurovirulent WNV NY99 strain 14 days after primary ZIKV or VSV infection. Viruses were back titrated to confirm inoculation doses. Animals were bled prior to infection, 5 and 13 days post-primary infection, and 5, 7 and 26 days post-WNV challenge (corresponding to 19, 21 and 40 days post-ZIKV infection, d.p.i.). Viral infections and sample collection were conducted as described.^36, 37, 38^

During the experiments, all animals were monitored daily and received water and food *ad libitum*. Those mice showing signs of disease were anesthetized and killed, as were all surviving animals at the end of the experiment (40 days after ZIKV infection).

### Immunological assays

Heat-inactivated sera (1:100 dilution) were assayed for anti-ZIKV antibodies by enzyme-linked immunosorbent assay (ELISA) as described^38, 39, 40^ using heat-inactivated viruses (WNV or ZIKV) produced in Vero cells as antigens. As positive controls, a WNV-specific mice sera pool^34^ and a ZIKV-specific mouse monoclonal antibody (mAb T39627), kindly provided by Dr R. Tesh (WRCEVA), were included in the assay. Specific antibody induction was represented as the fold increase of each sample sera absorbance_490_ over the absorbance_490_ of control sera from uninfected mice.

Measurements of serum IFN-α level were performed using the VeriKine Mouse Interferon Alpha ELISA Kit (PBL Assays Science, Piscataway, NJ, USA) following the recommendations provided by the manufacturer.

Plaque reduction neutralization tests (PRNT) were conducted on Vero cells with WNV and ZIKV strains MR766, FSS13025 and PA259459, using twofold serial sera dilutions.^39, 41^ Titers were calculated as the reciprocal of the serum dilution, diluted at least 1:20, which reduced plaque formation ≥90% (PRNT_90_) relative to samples incubated with negative control pooled sera.

### Virological assays

Viral RNA was extracted from the processed tissues and fluids using a Speedtools RNA virus extraction kit (Biotools, Madrid, Spain). ZIKV-RNA was quantified by real time quantitative reverse transcription PCR (qRT-PCR) as described,^42^ using a standard curve with previously titrated viruses.

### Statistical analyses

Data were analyzed using GraphPad PRISM 6 (GraphPad Software, La Jolla, CA, USA). Two-way ANOVA with Bonferroni post *t*-test and Kaplan–Meier survival analysis were performed. Asterisks in the figures denote statistically significant differences *P<0.05, **P<0.01 and ***P<0.001.

## RESULTS

### ZIKV infection protects mice against WNV challenge

**Figure 1:**
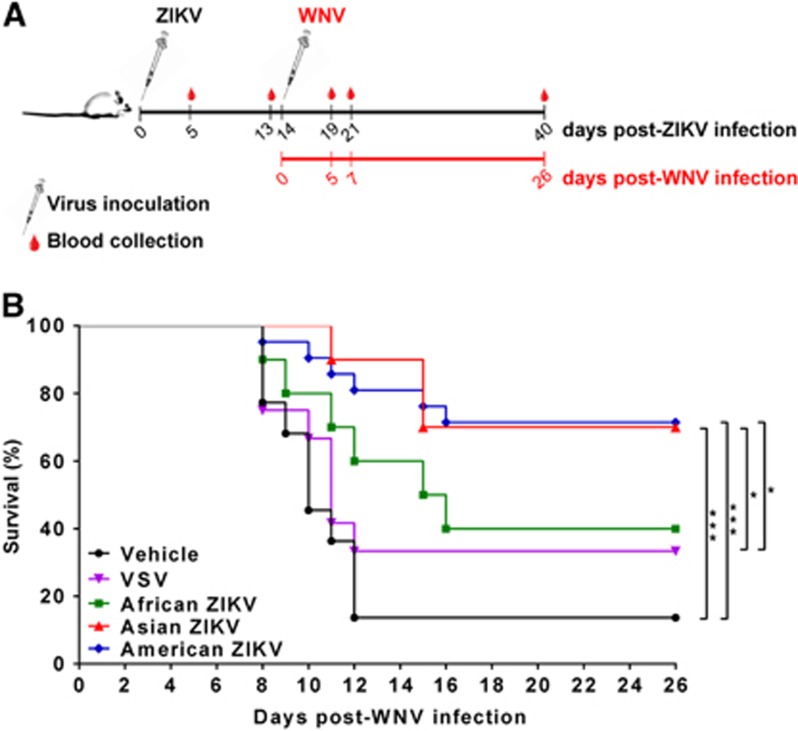
Protection conferred by ZIKV infection against subsequent WNV infection. (**A**) Experimental schedule representing the immunization timeline. Eight-week-old Swiss albino CD1 male mice (*n*=10–20/group) were infected i.p. with 5 × 10^5^ pfu/mouse of ZIKV (African, Asian or American strain), VSV, or culture medium (vehicle) as a negative control. Mice were subsequently infected intraperitoneally with WNV (10^4^ pfu/mouse) at 14 days post-ZIKV infection. (**B**) Survival rates in mice previously infected with the African, American, and Asian ZIKV strains, VSV or vehicle and challenged with WNV at 14 d.p.i. Statistically significant differences are indicated with asterisks *P<0.05, **P<0.01, ***P<0.001.

All mice survived i.p. ZIKV infection regardless of the origin of the infecting strain (African, Asian or American), and none showed signs of disease. Tissues (brains, spleens, and testicles) and fluids (urine and oral swabs) collected from ZIKV-infected, killed mice (six mice/group) at 5 d.p.i. were analyzed by qRT-PCR. Viral RNA was sporadically detected in a very limited number of samples (three brains, two spleens, one testicle and one urine sample), with titers ranging from 2 × 10^4^ to 2 × 10^9^ pfu/gram of tissue. To determine whether this infection was sufficient to elicit a protective immune response against other flaviviruses, mice infected with any of the ZIKV strains, with an unrelated virus (VSV), or with vehicle alone were challenged 14 days after infection with a highly virulent WNV strain (Figure 1A). Survival rates of 40%, 70% and 71% were recorded for WNV challenged mice initially infected with the African, Asian or American ZIKV strains, respectively, higher than the 33% and 14% recorded among VSV-inoculated and vehicle-inoculated animals, respectively (Figure 1B). Statistical analysis of survival curves confirmed that infection with the Asian or American strains conferred significant protection against a subsequent challenge with WNV compared to non-ZIKV-infected mice (vehicle and VSV-inoculated animals), confirming that the observed effect was specific for ZIKV. This was also observed when the mean survival times (MSTs) after WNV challenge were determined, as they were also higher in ZIKV-infected mice (>26 days for animals previously infected with Asian or American ZIKV, and 15.5 days for animals previously infected with African ZIKV) than in the control groups inoculated with vehicle alone (10 days) or with VSV (11 days).

### ZIKV infection enhances anti-WNV antibody induction

**Figure 2:**
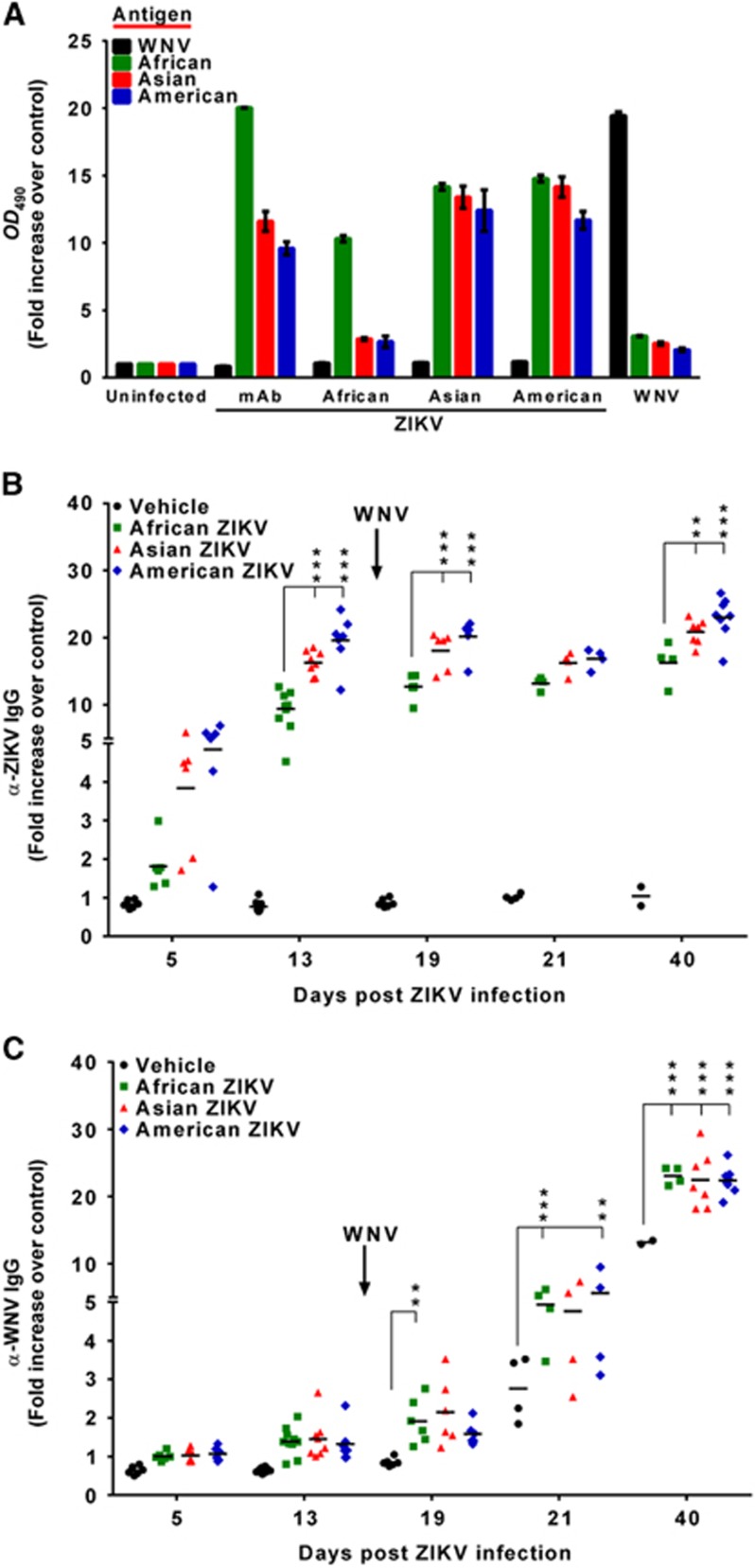
Induction of anti-ZIKV and anti-WNV IgGs in mice. (**A**) Assessment of reactivity and specificity of ELISAs for ZIKV and WNV. Plates were coated with ZIKV (African, American or Asian) or WNV antigens. Reactivity of sera pools of mice infected with the different ZIKV strains (13 d.p.i.) or control sera (WNV-specific sera pool or ZIKV-specific mAb) with the different antigens is represented as the fold increase of the sample *OD*_490_ over the *OD*_490_ of non-infected mice sera. Data are presented as the mean±SEM. (**B**) Anti-ZIKV or (**C**) anti-WNV IgGs in blood samples, collected at the days post-infection indicated in each case, were detected by an indirect ELISA using plates coated with heat-inactivated ZIKV or WNV, respectively. Solid lines represent the mean fold increase in absorbance of each group. Each point of the graph represents a single animal. Asterisks in panel B denote statistically significant differences among animals infected with the different ZIKV isolates. Asterisks in panel C denote statistically significant differences between animals previously infected with ZIKV and those only challenged with WNV (vehicle). *P<0.05, **P<0.01, ***P<0.001.

Because protection against flavivirus infection is primarily based on humoral responses,^25^ the antigenic relationships among the different viral strains were assessed. Cross-reactivity between ZIKV and WNV strains was addressed by testing a pool of sera from mice infected with the different viral isolates (13 days p.i.). For this, we utilized an in house developed ELISA based on inactivated whole-virus antigens produced from infected cell cultures. The ELISA was validated using a ZIKV-specific monoclonal antibody that showed good reactivity with the three ZIKV antigens, although it was higher with the African strain (Figure 2A), because this monoclonal antibody was produced against the African MR766 strain. Notably, whereas sera from mice infected with the Asian and American strains recognized the three ZIKV antigens in a similar manner, those from African infected mice mainly recognized their own specific antigen, as occurred with sera from WNV-infected mice (Figure 2A).

Therefore, individual mouse sera were assayed against their respective specific antigens. Some of the ZIKV-infected mice presented specific antibodies at 5 d.p.i., and all had detectable antibodies by day 13 p.i., which slightly increased after WNV challenge (Figure 2B). Remarkably, the African strain elicited a lower humoral immune response, as observed by a significantly lower level of antibodies in the sera of African ZIKV-infected mice compared to the other ZIKV-infected animals.

Interestingly, ZIKV infection enhanced specific WNV antibody production, because 5 days after WNV challenge (19 days post-ZIKV infection), specific anti-WNV antibodies were only observed in mice previously infected with ZIKV and not in those infected only with WNV (Figure 2C). Moreover, anti-WNV antibodies increased until the end of the experiment (40 days post-ZIKV infection, 26 days post-WNV infection), and were significantly higher in mice previously infected with ZIKV than in control mice infected only with WNV.

Considering the key protective role of neutralizing antibodies against flavivirus infections,^26, 27^ the neutralization capability of seropositive samples was tested against ZIKV and WNV isolates using PRNT (Table 1). Mice infected with the Asian or American strains neutralized the three ZIKV isolates, and those infected with the African strain only neutralized the homologous virus. Conversely, prior to WNV challenge, none of the sera nor the uninfected mouse sera neutralized WNV (Table 1). After WNV challenge, the neutralization capability of each serum was assayed against both their homologous ZIKV strain and WNV (Table 2). All tested sera were able to neutralize both viruses. None of the vehicle-inoculated mice infected with WNV presented neutralizing antibodies against ZIKV, and their PRNT_90_ titers against WNV were lower than those of ZIKV-infected mice (Table 2). To further confirm that the antibodies induced by WNV infection do not neutralize ZIKV, the reactivity of a panel of WNV-inoculated mice sera was tested 26 d.p.i. Although its mean PRNT_90_ titer against WNV was 740±137, they did not neutralize ZIKV, confirming data recorded at 7 d.p.i. Taken together, these results suggest that previous ZIKV infection could prime the mice to a faster response, enhancing subsequent anti-WNV neutralizing antibody production.

**Figure 3:**
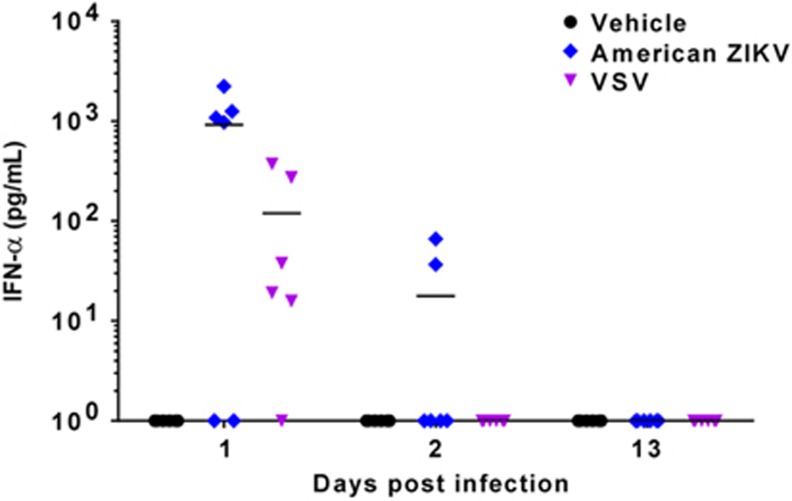
Induction of IFN-α in mice infected with ZIKV. Eight-week-old Swiss albino CD1 male mice (*n*=18/group) were infected i.p. with 5 × 10^5^ pfu/mouse of American ZIKV, VSV or culture medium (vehicle) as a negative control. A total of six mice per group were killed at 1, 2 and 13 days p.i., and IFN-α levels in serum were detected using a commercial assay. Solid lines represent the mean of each group. Each point of the graph represents a single animal.

Because the innate immune response contributes to virus clearance and a more rapid and effective humoral response,^43^ the production of interferon (IFN)-α in ZIKV-PA259459 infected mice was analyzed at early time points after ZIKV infection (1 and 2 d.p.i.), and prior to WNV challenge (13 d.p.i.). As a positive control, mice infected with VSV, in which the induction of IFN-α production has been documented,^44^ were also analyzed. Although both viruses induced detectable levels of circulating IFN-α at early time points (1 and 2 d.p.i. for ZIKV, and 1 d.p.i. for VSV), no detectable levels of IFN-α were observed at later times (13 d.p.i.) just prior to WNV challenge (Figure 3). These results indicate that the observed protection against WNV challenge in ZIKV-infected mice was not due to a sustained IFN-α response.

## DISCUSSION

Flaviviruses are expanding to new geographical regions where they previously did not represent a health problem, in part due to vector colonization of new areas. A current example of flavivirus spread is ZIKV, responsible for a recent epidemic in the Americas that quickly raised worldwide social and health concerns because of its possible association with severe neurological pathologies, such as GBS and microcephaly.^3, 4, 5, 6^

Currently, data about ZIKV pathogenesis and immunity remain scarce. We analyzed the development of a humoral response in immunocompetent mice infected with three different ZIKV isolates. In addition, considering a future eco-epidemiological scenario in which ZIKV will likely colonize new regions where WNV is already endemic, we studied the cross-reactivity of both flaviviruses. Because of the elevated seroprevalence of ZIKV during outbreaks (~75%)^45^ due to its high human-to-human transmission, and the relatively reduced incidence of WNV, the infection of which in humans is sporadic (~1%–2%),^46, 47^ we first infected mice with ZIKV and then challenged them with WNV. In this way, we recreated a possible eco-epidemiological scenario with rapid ZIKV spread and subsequent WNV infections. Our results showed that ZIKV-infected mice were protected against challenge with a neurovirulent WNV strain. Interestingly, survival rates of animals infected with the Asian or American strains were higher compared with animals inoculated with the African ZIKV. This effect was specific for ZIKV infection, because mice infected with the unrelated VSV were not significantly protected against WNV challenge. Because ZIKV strains are phylogenetically classified into two major lineages—one including the African strains, and the other the more recent Asian and American strains^3, 48^—our results suggest differences in immunogenicity between isolates from different lineages of ZIKV.

Antibody-mediated immunity is considered a major player in protection against flavivirus infections,^25^ including ZIKV.^26^ Moreover, cross-reactive antibodies can induce cross-protection against infection with related flaviviruses in some instances, even with quite low titers, but this is not always the case.^27^ Conversely, cross-reactive immunity is also associated with enhanced infection and disease outcome in DENV infections, mainly due to the ADE effect.^27^ Accordingly, there is evidence supporting ADE between DENV and ZIKV.^31, 32, 49, 50^ By contrast, our results showed that WN disease did not cause exacerbation in animals previously infected with ZIKV. Furthermore, animals infected with American or Asian ZIKV strains were partly protected against WNV challenge. In this sense, recent observations in immunocompromised mice injected with immune plasma from WNV- and DENV-positive donors suggest that cross-reactive antibodies can also protect against a challenge with ZIKV and that an ADE phenomenon was only observed within a reduced range of plasma concentrations.^51^ Although only one ZIKV serotype has been described,^52^ our results showed that while specific anti-ZIKV Asian and American sera recognized all three ZIKV antigens, sera from mice infected with the African strain mainly reacted with their homologous antigen. These results indicate a differential pattern of antibody induction within animals infected with different ZIKV isolates. Therefore, the African strain seemed to be less immunogenic, as shown by the lower levels of antibodies induced compared with the other two strains. After WNV challenge, an enhancement in the production of anti-WNV antibodies, including neutralizing antibodies, was recorded in mice previously infected with ZIKV compared to control mice infected only with WNV.

Polyprotein sequence analysis predicts the presence of potential N-glycosylation sites in some of the ZIKV proteins, including the envelope (E) protein, which is a major target for neutralizing antibodies.^3, 5^ The differences in immunization efficiency of the ZIKV strains used in this report may be due to distinct antigenicity or pathogenicity among isolates. For example, the African MR766 strain used in this study, representative of the African lineage, was derived from the original ZIKV isolated from a sentinel monkey in Uganda in 1947^13^ and corresponds to a highly mouse-adapted virus.^12, 13^ Consequently, sequence analysis of the MR766 strain used in this report confirmed that it exhibited a 4-amino acid deletion corresponding to the E protein 154 glycosylation motif found in many flaviviruses^48, 53, 54^ and that seems to play a role in the biology of ZIKV.^55^ By contrast, the two ZIKV strains from the Asian lineage (FSS13025 and PA259459) that correspond to non-mouse-adapted viruses isolated from infected humans in Cambodia (2010)^56^ and Panama (2015), respectively, display an intact N-glycosylation motif (VNDT) at the E protein. Consistent with these observations, differences in tissue tropism, pathogenic behavior, infectivity and cellular response between the strains of the African and Asian lineages were previously reported both *in vitro* and *in vivo* by other authors.^57, 58^ Thus, these differences may also contribute to the variation in the induction of the humoral response observed here.

Additionally, the activation of an adaptive immune response is related to active viral replication.^59^ Here, qRT-PCR analysis^42^ of the viral burden of tissues and fluids from ZIKV-infected mice killed 5 d.p.i. showed sporadic amplification in only a few of the infected mice (7/24), suggesting that viral replication is not a major player in the differences observed for protection between the ZIKV strains assayed. Although comparison between studies is difficult because mice strains, viral isolates, and time of sampling differ between them, these results are not very different from those described previously in other wild-type mice compared with immune compromised animals.^18, 19, 22^

In brief, no mortality or clinical signs of disease were recorded in any of the immunocompetent mice after i.p. infection with three ZIKV isolates of different geographical origin. However, our results suggest antigenic and immunogenic differences. Indeed, we demonstrated that, contrary to recent findings for ZIKV and DENV,^31, 32, 50^ WNV infection of ZIKV-infected mice did not induce ADE. Moreover, ZIKV infection elicited a protective immune response against WNV. It is worth mentioning that a study of protection against ZIKV by a previous WNV infection would be of interest. Herein, no cross-reactivity of WNV-induced antibodies against ZIKV was observed. However, considering that the cellular immune response could lead to some immunological cross-talk, resulting in protective immunity even in the absence of neutralizing antibodies, further studies should be performed. These findings may have implications in the eco-epidemiological scenario of regions not yet colonized by ZIKV where other flaviviruses circulate, and may be useful for the design of multi-flavivirus vaccines. However, further studies of the mechanism behind this protective response, including analysis of the cellular immune response, are required.

## Figures and Tables

**Table 1:** Induction of neutralizing antibodies in mice infected with ZIKV at 13 days post-ZIKV infection

		**Virus**		
**Mice sera**	**African ZIKV**	**Asian ZIKV**	**American ZIKV**	**WNV**
**ZIKV**				
African	125	Negative	Negative	Negative
Asian	80	135	140	Negative
American	75	60	50	Negative
				
Vehicle	Negative	Negative	Negative	Negative

Abbreviations: West Nile virus, WNV; Zika virus, ZIKV.

Values represent PRNT_90_ titers of pooled mice sera. Negative denotes PRNT_90_ value<20.

**Table 2:** Induction of neutralizing antibodies in mice infected with ZIKV and challenged with WNV at 21 days post-ZIKV infection (7 days post-WNV challenge)

		**Virus**		
**Mice sera**	**African ZIKV**	**Asian ZIKV**	**American ZIKV**	**WNV**
**ZIKV**				
African	163±96	NT	NT	320±59
Asian	NT	148±36	NT	254±186
American	NT	NT	166±93	175±39
				
Vehicle	Negative	Negative	Negative	88±53

Abbreviations: West Nile virus, WNV; Zika virus, ZIKV; not tested, NT.

Values represent the mean PRNT_90_ titers of mice sera for each group±SEM. Negative denotes PRNT_90_ value<20.
